# *Piper sarmentosum* Leaves Aqueous Extract Attenuates Vascular Endothelial Dysfunction in Spontaneously Hypertensive Rats

**DOI:** 10.1155/2019/7198592

**Published:** 2019-08-14

**Authors:** Fatimatuzzahra Hashim Fauzy, Maizura Mohd Zainudin, Hidayatul Radziah Ismawi, Taher F. T. Elshami

**Affiliations:** Department of Basic Medical Sciences, International Islamic University Malaysia, Kuantan 25200, Malaysia

## Abstract

*Piper sarmentosum* is a tropical plant in Southeast Asia known for its traditional use in curing various ailments including hypertension. Previous research works have provided evidence for the herb's antihypertensive property. However, the exact mechanisms involved are still in question. The present study investigated the effects of *Piper sarmentosum* leaves aqueous extract (PSAE) treatment on vascular endothelin system in spontaneously hypertensive rats (SHRs). Four groups of SHRs were treated for 28 consecutive days, with negative and positive control groups receiving distilled water and 3 mg/kg perindopril, respectively. Another two groups are the treatment groups, which received PSAE and combination of 1.5 mg/kg perindopril and PSAE. Weekly measurements of blood pressure showed that PSAE significantly reduced the systolic, diastolic, and mean arterial pressures (*P* < 0.05) of the rats. PSAE also increased mesenteric artery nitric oxide (NO) level (*P* < 0.05) and reduced endothelin-1 (ET-1) level (*P* < 0.05) in the treatment groups. Our results demonstrate that oral administration of PSAE reduced blood pressure in SHRs by reducing the ET-1 level while increasing NO production.

## 1. Introduction

Hypertension remains a health burden worldwide as it is one of the key risk factors for cardiovascular diseases, the biggest contributors of global mortality [1]. Being the leading preventable cause of premature death worldwide, the attention hypertension got over the past decades until today is plethoric. However, despite established studies of antihypertensive medications, substantial hurdles still remain in overcoming the disease [2–4]. One major factor that contributes to this problem is the complexity of the disease itself. The pathophysiology of hypertension involves de trop interplay between renal, neural, cardiovascular, and endocrine factors modulated by genetic and environmental factors. This complex polygenic disorder occurs as a result of additive effects of multiple variant genes working en masse and in concert with the necessary environmental conditions to elevate blood pressure [5–9].

One of the mechanisms responsible for elevated blood pressure in hypertension is the impairment of endothelial physiological function, otherwise known as endothelial dysfunction [10–12]. Endothelial dysfunction is characterized by an imbalance between endothelial-derived vasodilating and vasoconstricting factors which are nitric oxide (NO) and endothelin-1 (ET-1), respectively, by which the former is downregulated and vice versa [13]. Failure of the physiological balance between these two molecules breaks down the homeostasis of the vascular beds, abetting the pathological process of the vascular diseases. This imbalance occurs via reduced NO synthesis, reduced NO bioavailability, or antagonism of NO by endothelium-derived contracting factors such as ET-1 [12].

ET-1 is one of the three endothelins expressed in humans, secreted by endothelial cells in blood vessels and in many other cells of the body. This main endothelin is a potent vasoconstrictor [14] and, via ET_A_ receptor activation, causes various actions in the kidney. When administered on a long-term basis, it results in an increase of blood pressure and contributes to the development of hypertension [15]. It also stimulates oxidative stress by causing upregulation of the nicotinamide adenosine dinucleotide phosphate (NADPH) oxidase subunits, increasing superoxide [16]. These characteristics of ET-1 make it a key regulator in hypertension and a biomarker for endothelial dysfunction.

*Piper sarmentosum* Roxburgh (*Piperacea*) is a herbaceous plant familiar in the Southeast Asian region. It has long been used traditionally as a herbal medicine to treat diabetes, hypertension, and joint aches [17], toothache, coughing, pleurisy, fever, headache [18], feet dermatomycoses, and indigestion [19]. Extensive studies have been done for its pharmacological activities such as antihypertensive [20, 21], anti-inflammatory [22, 23], antiatherosclerosis [24, 25], antiangiogenic [26], hypoglycemic [27], antifungal [28, 29], antibacterial [30, 31], and antiprotozoa [32]. This plant also exhibits high antioxidant property [17, 33, 34]. The high antioxidant activity exhibited by this plant helps to balance reactive oxygen species (ROS) formation, thus alleviating endothelial oxidative stress, increasing NO bioavailability in human umbilical vein endothelial cells (HUVECs) [34].

Recent study with PSAE showed that administration of the extract lowers blood pressure in SHRs, affecting diastolic blood pressure (DBP) more than systolic blood pressure (SBP) [20]. Supplementation with *Piper sarmentosum* in this study also demonstrated an increased NO level in SHRs, promoting vascular relaxation. However, whether endothelin system is involved in the mechanism is still yet to be determined. Furthermore, although involvement of resistance artery in blood pressure regulation is pivotal, there is no report about the effect PSAE has on them. In the present study, we investigated the efficacy of PSAE in maintaining vascular endothelin system homeostasis as one of the mechanisms responsible for lowering blood pressure in SHRs.

## 2. Materials and Methods

### 2.1. Plant Material

Fresh PS leaves were collected from a thicket in Kuantan, Pahang, Malaysia (GPS coordinate: 3.8098666979082325, 103.31667760157472), on 10th and 11th of October 2016 at 9.30 to 11.30 in the morning. The specimen sample was authenticated by Dr. Shamsul Khamis, a botanist at the National University of Malaysia (UKM) Bangi Herbarium, and deposited in the Herbarium, Kulliyyah of Pharmacy, IIUM Kuantan, Malaysia (voucher specimen number: PPIUM 0239-2 [VS-4]).

### 2.2. Preparation of *Piper sarmentosum* Leaves Aqueous Extract

Fresh *Piper sarmentosum* leaves were washed thoroughly with tap water and then cut and dried under shade at ambient temperature for three days. The leaves were then further dried in an oven at 40°C for four hours until they became brittle. The dried leaves were triturated until they turned into coarse powder and kept in hermetically sealed jars at 4°C until further processing. The powdered dry leaves were then extracted at the General Laboratory of Central Research and Animal Facility (CREAM), Kulliyyah of Science, IIUM, Kuantan in Pahang, Malaysia. An amount of 100 grams of the powdered leaves were added to 900 ml of distilled water and heated at 80°C for three hours. The concentrated plant extract was then cooled and filtered, before lyophilised by a freeze-drier. Finally, the powdered form extract was stored at 4°C until use.

### 2.3. Experimental Animals

Male SHRs aged 11 weeks old with initial 250 ± 10% g body weight obtained from Animal Research and Service Centre (ARASC), University of Science Malaysia (USM) Kubang Kerian, Kelantan, Malaysia, were used in this study. The rats were kept singly in polypropylene cages under standard laboratory conditions of 24 ± 4°C temperature, with relative humidity of 55 ± 10%, in 12 hour light dark cycle. Adequate cross ventilation was provided, and they were given standard commercial dry pellet (Gold Coin Sdn. Bhd.) and distilled water ad libitum.

After one week acclimatization with weight increment, a total of 24 SHRs were randomly and equally divided into four groups: negative control group which received an amount of vehicle (distilled water) according to PSAE dose, not exceeding 2 ml/100 g body weight/day; positive control group which received 3 mg/kg/day perindopril (Servier Malaysia Sdn. Bhd); PSAE group which received 500 mg/kg PSAE; and PSAE + perindopril group which received 500 mg/kg PSAE and 1.5 mg/kg/day perindopril. Baseline blood pressure was measured and recorded before treatment was started. Treatment was given for 28 consecutive days. The formulated dose of PSAE and period of treatment were based on studies by Mohd Zainudin et al. [20]. The treatment materials were prepared into solutions beforehand and were administered intragastrically via oral gavage. The rats' blood pressure was measured weekly during the treatment period. On day 29, the rats were dissected, and mesenteric arteries were harvested. The samples were analysed for determination of the vascular ET-1 and NO levels. All procedures were conducted in accordance with animal ethics and protocol from the guidelines of the National Institutes of Health Guide for the Care and Use of Laboratory Animals [35] and approved by IIUM Institutional Animal Care and Use Comitte (IACUC-IIUM), Malaysia, with the approval number IIUM/IACUC Approval/2016/(11)(72).

### 2.4. Determination of Blood Pressure and Heart Rate

The rats' blood pressure (BP) was measured in the morning between 8 and 11 am weekly after 22–24 hours of administration of vehicle or treatment substances. They were measured in conscious, prewarmed, and restrained rats using the noninvasive technique by a plethysmographic tail-cuff method by the CODA™ noninvasive blood pressure (NIBP) system (Kent Scientific Corporation, USA). Thirteen determinations were made in every session of BP measurement, and the mean of three values within 15 mmHg was taken as the BP level.

### 2.5. Dissection and Preparation of Mesenteric Artery

**Figure 1:**
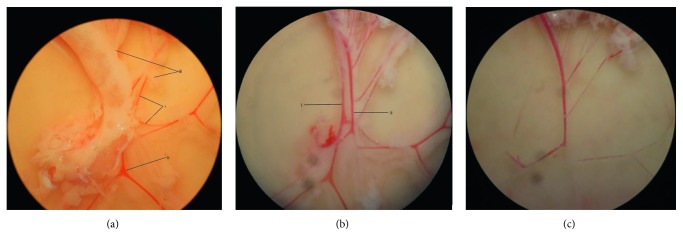
Images of mesenteric vessels under dissecting microscope. (a) Before removal of connective tissues and nerve bundle with (i) artery, (ii) vein, and (iii) adhering fat visible. (b) After removal of most connective tissue and nerve bundle. (i) The artery located under (ii) the veins appeared “whiter” than the latter. (c) The cleaned arteries.

The rats were anaesthetized intravenously with Ketamine + Xylazine + Zoletil® (KTX) cocktail and dissected. The entire small intestinal mesenteric bed, still attached to the small intestines, was then harvested and submerged into ice-cold PBS. The SHRs were then euthanized by cardiac removal. Gentle blunt dissection was done on the mesenteric arcade to remove nerve bundles, connective tissues, mesenteric vein, and adherent fat from the mesenteric artery on bone wax-coated Petri dish on ice (Figure 1). Finally, the cleaned mesenteric artery was snap-frozen in liquid nitrogen and kept at −80°C until further analysis.

### 2.6. NO Measurement

Mesenteric artery NO level was determined by measuring nitrite (NO_2_^−^) concentration using QuantiChrom™ Nitric Oxide Assay Kit (D2NO–100) obtained from i-DNA Biotechnology Sdn Bhd, Malaysia. The kit applies colorimetric assay according to the improved Griess method.

### 2.7. ET-1 Measurement

The concentration of ET-1 in mesenteric artery was determined using a rat ET-1 ELISA kit (Elabscience®, USA).

### 2.8. Statistical Analysis

Results were expressed as mean ± standard error of mean (SEM). A one-way repeated measures analysis of variance (ANOVA) was run to compare mean blood pressure between study groups during treatment period while Bonferroni post hoc test was performed to test the significance of mean difference between the groups. Meanwhile, the statistical mean difference of NO and ET-1 level between the four study groups were compared by one-way ANOVA with subsequent post hoc Tukey's HSD test. *P*-values of less than 0.05 were considered to be statistically significant.

## 3. Results

### 3.1. PSAE Reduces Blood Pressure but Not Heart Rate in SHRs

**Figure 2:**
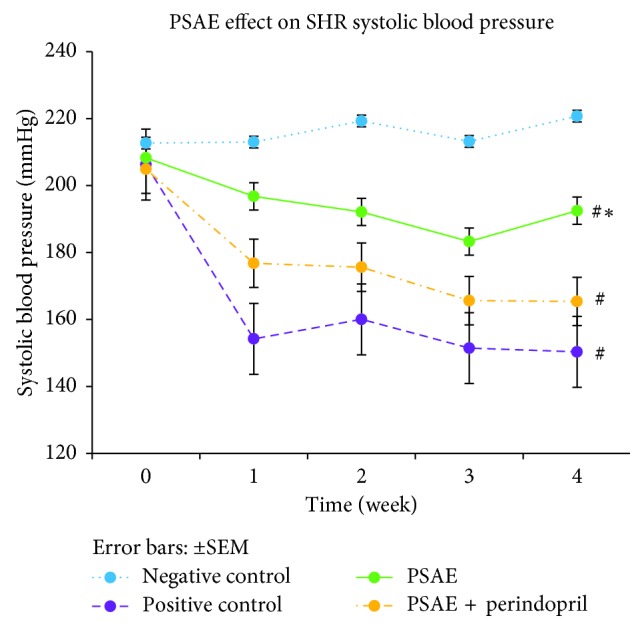
Weekly evolution of systolic blood pressure obtained by the tail-cuff method. Negative control = nontreated SHRs. Positive control = SHRs treated with perindopril. Values are expressed as mean ± SEM with *n* = 6. ^#^Significant mean difference with negative control group (*P* < 0.05); ^*∗*^significant mean difference with both positive control and PSAE + perindopril combination group (*P* < 0.05).

**Figure 3:**
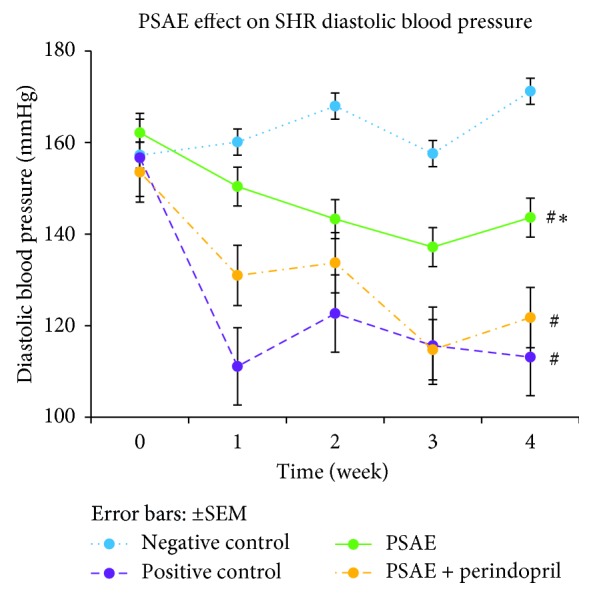
Weekly evolution of diastolic blood pressure obtained by the tail-cuff method. Values are expressed as mean ± SEM with *n* = 6. Negative control = nontreated SHRs. Positive control = SHRs treated with perindopril. ^#^Significant mean difference with negative control group (*P* < 0.05); ^*∗*^significant mean difference with both positive control and PSAE + perindopril combination group (*P* < 0.05).

**Figure 4:**
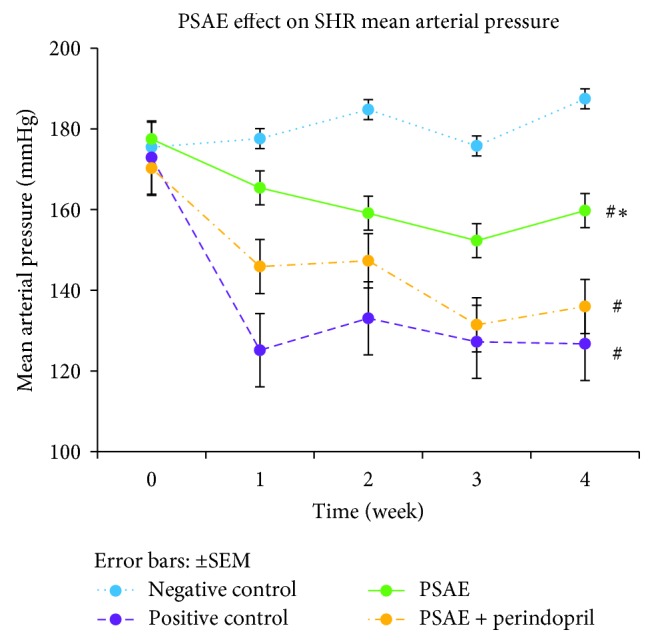
Weekly evolution of mean arterial pressure obtained by the tail-cuff method. Values are expressed as mean ± SEM with *n* = 6. Negative control = nontreated SHRs. Positive control = SHRs treated with perindopril. ^#^Significant mean difference with negative control group (*P* < 0.05); ^*∗*^significant mean difference with both positive control and PSAE + perindopril combination group (*P* < 0.05).

After the treatment was started, all of the treated groups displayed significantly lower (*P* < 0.05) mean SBP, DBP, and MAP as compared to the negative control group. However, among the treated groups, PSAE-treated rats showed significantly higher mean SBP, DBP, and MAP compared to rats in the PSAE + perindopril-treated group and positive control group. In the meantime, no significant differences of mean SBP, DBP, and MAP were observed between the PSAE + perindopril combination-treated group and the positive control group (Figures 2–4).

**Figure 5:**
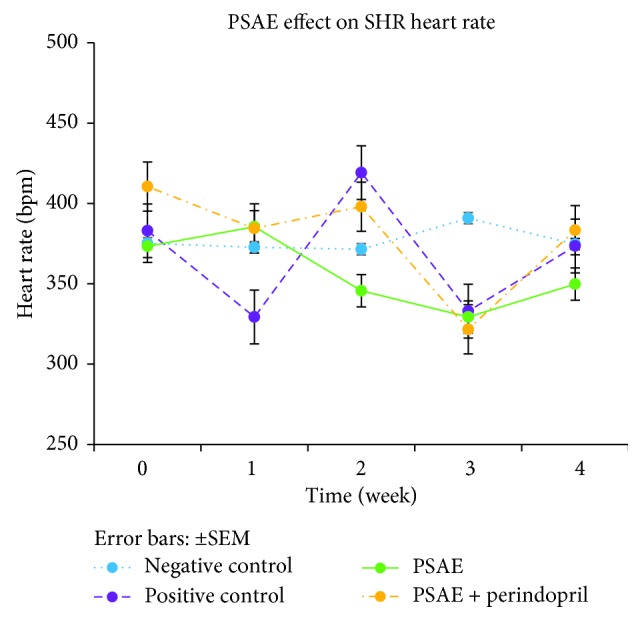
Mean of SHRs heart rate obtained by the tail-cuff method. Values are expressed as mean ± SEM with *n* = 6. Negative control = nontreated SHRs. Positive control = SHRs treated with perindopril.

In this study, mean heart rate of all groups demonstrated inconsistent values throughout the study period. There was no statistically significant difference (*P* < 0.05) in the heart rate recorded among all of the groups during treatment period (Figure 5).

### 3.2. Effects of PSAE on NO Level in Mesenteric Arteries

**Figure 6:**
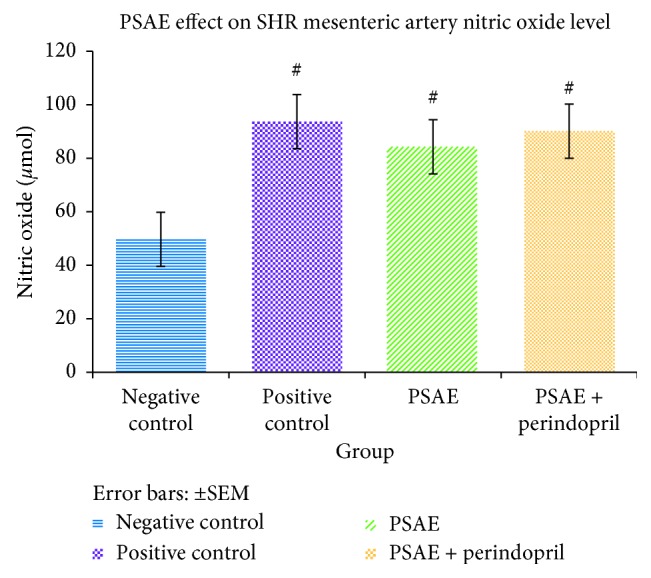
Effect of PSAE and PSAE + perindopril combination on the nitric oxide (NO) level of spontaneously hypertensive rat (SHR). Values are expressed as mean ± SEM (*P* < 0.05), with *n* = 6. A = PSAE-treated group, B = PSAE + perindopril-treated group. Negative control = untreated SHRs, Positive control = perindopril-treated SHRs. #Significant mean difference with negative control group (*P* < 0.05).

In present study, SHRs administered with PSAE, PSAE + perindopril, and perindopril orally for 28 days had a significantly higher (*P* < 0.05) NO level in the mesenteric arteries as compared to the untreated group. The PSAE-treated group showed the lowest mean NO level among the treatment groups. However, there was no significant difference of mean NO level between the PSAE-treated group with both positive control group and the PSAE + perindopril-treated group (Figure 6).

### 3.3. Effects of PSAE on ET-1 Level in Mesenteric Arteries

**Figure 7:**
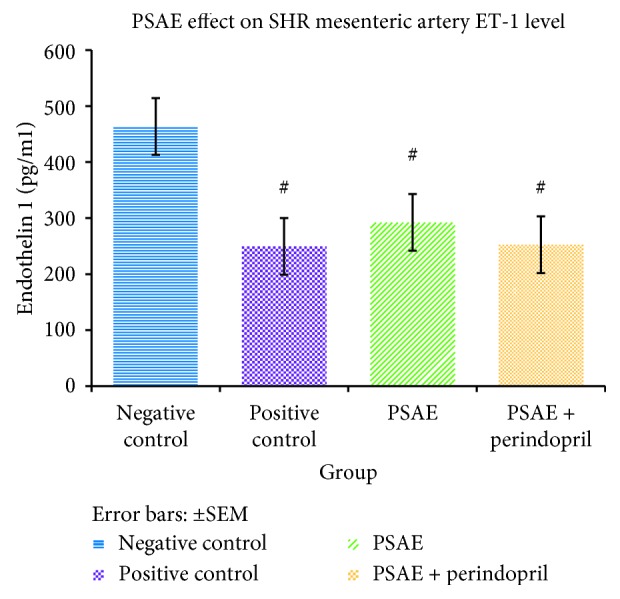
Effect of PSAE and PSAE + perindopril combination on the endothelin-1 (ET-1) level of spontaneously hypertensive rat (SHR). Values are expressed as mean ± SEM (*P* < 0.05), with *n* = 6. Negative control = untreated SHRs, Positive control = perindopril-treated SHRs. #Significant mean difference with negative control group (*P* < 0.05).

Both groups receiving PSAE and PSAE + perindopril, as well as the positive control group, recorded significantly (*P* < 0.05) lower mesenteric arteries ET-1 concentration than the negative control group. Among the treatment groups, mean ET-1 concentration was highest in the group receiving PSAE. Nevertheless, there was no statistically significant difference of mean ET-1 level between the PSAE-treated group with both the positive control group and PSAE + perindopril-treated group (Figure 7).

## 4. Discussion

Endothelium is a squamous, single-cell lining membrane covering the inner wall of vessels. Once thought to only function as a barrier separating the circulating blood and vascular smooth muscle cells (VSMC), endothelium is now considered as having an important role in modulating vascular function. Endothelial cells synthesize and release a number of essential factors that are involved in maintaining the homeostatic balance of blood vessels. Any imbalance of these mediators' bioavailability leads to vascular diseases such as hypertension [36]. Two of the mediators are NO and endothelin. These two mediators have a close relationship in maintaining the balance in endothelial functions in which the disequilibrium will cause endothelial dysfunction [37].

Endothelial dysfunction, which refers to an impairment in its vasodilatory capacity, plays a crucial role in pathogenesis of hypertension [12]. The characteristic imbalance between endothelium-dependent vasodilation and vasoconstriction in endothelial dysfunction occurs through reduced NO synthesis, reduced NO bioavailability, or antagonism of NO by endothelium-derived contracting factors. One of the causes that can reduce synthesis of NO is decreased endothelial nitric oxide synthase (eNOS) activity caused by competitive inhibition of eNOS by asymmetric dimethylarginine (ADMA). Meanwhile, reduced bioavailability of NO can be caused by reactive oxygen species (ROS) which convert NO to peroxynitrite [12, 38]. Diminished NO bioavailability caused by the oxidative stress causes unabated actions of ET-1 leading to vasoconstriction and vascular remodelling, resulting in increased blood pressure. Results from our study provide more evidence of antihypertensive property of PSAE and the mechanisms that may be responsible. Oral administration of PSAE reduces blood pressure and may improve resistance arteries endothelial function by increasing NO bioavailability, thus decreasing ET-1 in SHRs.

SHRs are known to display many characteristics of the human essential hypertension [39]. The increase in blood pressure and peripheral resistance in SHRs is preceded by vascular oxidative stress, which results in endothelial dysfunction [40]. Reduction of blood pressure in our study may partly be caused by debilitation of the endothelial dysfunction present in the animals.

Apart from having a role as a potent vasoconstrictor, ET-1 also upregulates NADPH oxidase (NOX) subunits, which are a major source of ROS in the cardiovascular system. Thus, ET-1 increases superoxide anion, stimulating oxidative stress [41]. However, ET-1 pathway is antagonized by NO through reduction of the preproendothelin-1 (ppET-1) mRNA via downregulation of ppET-1 gene transcription and through endothelial cGMP-dependent mechanism [42, 43]. NO also decreases the duration of interaction between ET-1 and its receptors, as well as inhibiting ET-1 signaling at the VSMC calcium signaling level [44].

Accordingly, an increased NO level will mitigate ET-1 actions and thus rehabilitate endothelial function. On the other hand, a decrease in the NO level, which occurs during hypertension, will pathologically lead to an increased ET-1 level, causing endothelial dysfunction. The action of NO in antagonizing ET-1 is proven by the present study, in which the levels of ET-1 in the resistance vessels of the rats in the treated groups were significantly lower than that in the negative control group. Meanwhile, the NO levels of the same mesenteric arteries in the PSAE-treated and PSAE + perindopril-treated rats were significantly higher as compared to the negative control group. The effect was comparable to the rats in positive control group, which were treated by perindopril, a long-acting angiotensin converting enzyme (ACE) inhibitor. This drug works on renin-angiotensin-aldosterone system (RAAS) by inhibiting ACE, which is responsible for converting angiotensin I into angiotensin II (ANG II), a potent vasoconstrictor. ANG II also stimulates vasopressin and aldosterone secretion which increase water and sodium retention. These actions of ANG II increase both preload and afterload, subsequently increasing blood pressure [45].

Reduction of endothelial NO bioavailability is closely associated with an overall increase in ROS [46, 47], which may explain the reason why the mechanism of endothelial dysfunction which contributes to hypertension is also strongly related with oxidative stress. Hence, the ability of PSAE in increasing NO bioavailability may be caused by its capacity of maintaining the redox balance resulting from its rich antioxidant content.

Furthermore, several chemical constituents in *Piper sarmentosum* (PS) such as naringenin [17], quercetin [48], sesamin [49], rutin, and vitexin [50] have been reported to protect blood vessels and might be able to convalesce vascular injury. Quercetin, a flavonoid compound, has been proven to exhibit cardioprotective effects in prehypertensive men and women by improving endothelial function and reducing inflammation [51]. It is able to improve endothelial function by preventing ET-1-induced vascular superoxide anion production. This action is accomplished by reduction of p47phox overexpression, thus reducing subsequent increased NOX activity and uncoupled eNOS [52]. A study by Loke et al. in 2008 suggested that quercetin and epicatechin can acutely improve endothelial function by modulating the circulating concentrations of NO and ET-1 which was possibly exerted through inhibition of NOX and activation of eNOS [53]. Another flavonoid in PS, vitexin, possesses antihypertensive property by causing relaxation of vascular contraction regardless of endothelial function, by inhibiting phorbol ester-induced increases in pERK1/2 levels [54]. Vitexin has also been reported to be a hypotensive compound by having diuretic effects attributed to the inhibition of sodium-chloride-symporter system in the renal distal tubule, instead of having direct vasodilation effect [55]. Rutin, which is also one of the flavonoids in PS may improve endothelial function by augmenting NO production through inducement of eNOS gene expression, protein synthesis, and activity in oxidative stress-induced HUVEC [49]. Another chemical constituent contained in PS, sesamin, was reported to induce NO and inhibit endothelin converting enzyme (ECE) gene expression through NO signaling. Sesamin also acts as an antioxidant, decreasing ROS and inducing NOS gene expression [56]. However, further studies are needed to determine whether the antihypertensive effect of PSAE is due to their original composition and the synergistic action of several antioxidant compounds contained in the plant or due to a unique active compound.

On the other hand, for blood pressure, our study found that PSAE did reduce blood pressure but showed no superior effect as compared to perindopril. The rats treated with combination of half dose of perindopril and PSAE however displayed lower blood pressure during treatment period comparable to those receiving full dose of perindopril. Nonetheless, whether the effect had been solely contributed by addition of PSAE needs further investigation. The dose of perindopril used in the combination therapy during this study was halved to investigate the effect of PSAE cotreatment with a lowered treatment dose of conventional therapy. Optimizing drug dosage permits the benefits of minimizing drug wastage incidence and increasing compliance, holding potential for enhancing therapeutic outcomes while concomitantly decreasing the incidence of adverse drug events, as well as reducing patient healthcare cost [57].

Although the exact mechanism(s) regarding how PSAE lowers blood pressure is not fully understood yet, results from our study suggested that attenuation of vascular endothelial dysfunction due to NO bioavailability augmentation might be substantially responsible. This is evident when perindopril, which works on RAAS, lowered blood pressure more than PSAE did while the same rats of all treated groups had statistically comparable ET-1 and NO levels at the end of the study. Such inference is plausible considering the complexity of essential hypertension pathogenesis that involves interaction of multiple organ systems and various mechanisms.

## 5. Conclusions

This study demonstrated that the antihypertensive effect of orally administered PSAE in SHRs may be due to its ability to ameliorate endothelial dysfunction by increasing the resistance artery NO level and reducing the resistance artery ET-1 level. The effect may be attributed to high antioxidant property of the plant, though more thorough study is needed to prove it. This present study also showed that the effects of PSAE given once daily may not be superior to those of full-dose perindopril. Thus, a further study that analyses a “bis in die” (BD) dose of PSAE on the SHRs is needed to elucidate whether PSAE has a comparable antihypertensive effect to a full-dose perindopril. Further study by combining the plant extract with full dose of perindopril is also needed to evaluate whether the plant addition exerts more beneficial effects or not. This will confirm the plant synergistic effect with perindopril and its ability as adjunctive treatment. In addition, meta-analysis trials with large sample size and excellent methodological qualities will help to provide a confirmed conclusion of the effectiveness and safety of PSAE as adjunctive treatment for essential hypertension. Finally, a study on long-term effect of prolonged PSAE ingestion, as well as a clinical study, will also be needed in order for PSAE to be used in humans.

## Data Availability

The data used to support the findings of this study are available from the corresponding author upon request.
